# Applicant Selection for Anesthesiology Residency Programs in Saudi Arabia

**DOI:** 10.7759/cureus.30071

**Published:** 2022-10-08

**Authors:** Mohammed Almatrodi, Fatma Aldammas, Adel Alqarni, Faris Alwarhi, Abdulelah Alotaibi, Amal Alqarni, Raghad Bedaiwi

**Affiliations:** 1 College of Medicine, King Saud University, Riyadh, SAU; 2 College of Medicine, King Abdul-Aziz Medical City, Riyadh, SAU; 3 College of Medicine, King Khalid University Hospital, King Saud University, Riyadh, SAU; 4 College of Medicine, Princess Nourah Bint Abdul Rahman University, Riyadh, SAU

**Keywords:** saudi arabia, knowledge, cognitive factors, academic, anesthesiology

## Abstract

Background

Anesthesiology is a medical specialty that involves pre, intra, and postoperative surgical and medical procedures; it is a profession shaped by the clinician’s medical knowledge and manual dexterity. To date, very few studies have addressed the selection criteria and factors associated with the applicant selection process for anesthesia residency programs in Saudi Arabia.

Objectives

We aimed to define the criteria, factors, and guidelines for candidate selection in anesthesia residency programs in Saudi Arabia.

Methodology

This was a cross-sectional study conducted using electronic questionnaires that were distributed to anesthesiology program directors in Saudi Arabia via email. The questionnaire was divided into six sections, and each section included various parameters such as demographic data, cognitive/academic activities, non-cognitive/non-academic activities, individual qualities, and red flags or negative factors of the individual. The participants rated each parameter, and the collected data were analyzed for statistical significance (p≤0.05).

Results

A total of 28 programs were included in this survey. All 14 parameters associated with individual qualities were found to be significantly important for applicant selection (p≤0.05). Except for delayed entry into residency after graduation, all 12 parameters associated with red flags or negative characteristics of individuals were significant for candidate selection (p≤0.05).

Conclusion

The results showed that academic/cognitive factors and non-academic/non-cognitive factors, along with the individual characteristics of the applicant, were given priority when selecting candidates for anesthesiology residency programs in Saudi Arabia.

## Introduction

There are several different pathways for future physicians to follow when pursuing a medical specialty [1]. Students begin to consider suitable medical specialties to pursue early on in medical school. Moreover, as students progress through medical school, they get to experience and explore multiple specialties such as surgery, internal medicine, pediatrics, obstetrics, and gynecology [2]. However, cultural, societal, and prestige factors may influence students’ decisions regarding their medical specialty [3].

Anesthesiology comprises of pre, intra, and postoperative surgical and medical procedures. It involves pain management through the administration of anesthetic medications required in the procedures. Furthermore, an anesthesiologist’s profession is shaped by their medical knowledge and hand skills [4]. Since 1956, anesthesiology has grown to become one of the foundations of supporting medical disciplines for contemporary medical services in Saudi Arabia; prior to this era, the surgeon would operate on patients who were fully awake [5]. In Saudi Arabia, the anesthesiology residency program lasts five years after completing six years of a Bachelor of Medicine, Bachelor of Surgery degree (MBBS) and is aimed at training anesthetists to become capable of and skilled in performing general and regional anesthesia procedures in addition to performing a comprehensive preoperative assessment of patients and training in subspecialties such as acute and chronic pain management [6].

Application to the anesthesiology residency program is competitive, and several factors can influence the decisions of the directors of residency training programs when reviewing a candidate’s application [6]. Understanding these factors may be critical for those considering the anesthesiology program in order to improve their chances of acceptance by attending at least eight to 10 interviews. In addition, these factors may also help program directors in selecting the best and most qualified candidates who are capable of excelling in all aspects related to the anesthesiology profession and the scientific research associated with it. According to a study conducted in Saudi Arabia in 2009, the strongest predictor of the selection process was the interview score [7].

Another study found that anesthesiology residency acceptance was primarily related to US medical school attendance and US Medical Licensing Examination step two scores; age and gender bias played a role in the selection process [8]. Increased requirements for more research related to this issue have been recognized, including the urgent need to specifically target medical students and academic program directors in Saudi Arabia. Higher scores in the United States Medical Licensing Exam (USMLE) step one and step two are associated with successful admission to anesthesiology training programs [9]. This literature suggests that anesthesiology residents are all physicians. Different universities worldwide have varying criteria for admission to anesthesiology residency programs based on the needs and requirements of the resident population. To date, several criteria have been discussed in the literature. However, most studies have been conducted in the United States. Countries in the Gulf Cooperation Council (Bahrain, Kuwait, Oman, Saudi Arabia, Qatar, and the United Arab Emirates) have contributed very little to this area of study. In the United States, the announcement that the USMLE step one scores will be replaced with a pass/fail status, including the increasing competition in obtaining an interview invitation, has made it more challenging and difficult for the decision committee to review all applications [10]. In Saudi Arabia, the factors associated with residency selection are broad, constantly changing, and have not been sufficiently examined [6]. To the best of our knowledge, there are no specific guidelines regarding the residency selection process in Saudi Arabia. The parameters used to evaluate the applicants remain unclear, whether it’s based on application, interview scores, or their individual qualities, characteristics, and other various assessment measures. Candidates must attend several match interviews before receiving match status notifications via email. Applicants can also access their match status via mobile device in the registration, ranking, and results section. In this study, we aimed to investigate the precise standards that lead to a successful and ideal residency matching process in Saudi Arabia and to maximize the acceptance opportunities of candidates through the understanding of these standards.

## Materials and methods

Participant selection

This study was approved by the Institutional Review Board Committee of the College of Medicine at King Saud University. Informed consent was obtained from the staff members included in this study.

This cross-sectional study was conducted with 28 participants between October 2021 and December 2021. The participants included current anesthesiology program directors, former anesthesiology program directors, residency training program committee consultants, and chief residents in Saudi Arabia. The participants were approached by the research team via e-mail and were asked to fill out an electronic survey after being assured that their data would remain anonymous and would only be used for research purposes. The electronic survey covered important aspects and factors that may influence residency training program directors when selecting an anesthesia resident. It was designed by the investigators and reviewed for comprehensiveness by two expert anesthetists. The survey was piloted on five Arabic-speaking volunteers with non-medical backgrounds to check for clarity, and necessary adjustments were made based on their responses.

Construction of the survey

The survey was divided into six sections. The first section enquired about demographic data, including age, sex, the region of Saudi Arabia the participant had worked in in the last five years, level of training, duration of experience in practicing anesthesia, and whether the participant had been involved in selecting residents in the last five years. The second section focused on cognitive/academic measures, including medical school grade point average (GPA), pre-clinical or pre-clerkship grades, clinical or clerkship grades, medical student performance record or Dean’s letter, academic awards, research experience, publications, master’s or doctorate degree awarded, reference letters, elective experience in anesthesiology, medical school reputation, and undergraduate/pre-med GPA.

The third section covered non-cognitive/non-academic measures such as extracurricular activities in medical school, international/community services, leadership positions held, Casualty Actuarial Society (CAS)-is a leading international organization for credentialing and professional education/American Society of Anesthesiologists (CAS/ASA) membership, personal statements, conduct on social media, applicant contact with program directors/colleagues, and work experience.

The fourth section focused on individual qualities, including professionalism, leadership skills, critical thinking skills, communication skills, interpersonal skills, maturity, motivation, confidence, altruism, attitude, dexterity, quality of questions and answers, and composure during the interview.

The fifth section focused on negative factors or red flags during applicant evaluation, such as receiving disciplinary action in medical school, delayed entry into residency after graduation, failure in a required clerkship rotation, failure in a pre-clinical course, absence of extracurricular activities, poor literacy or communication skills, extended time needed to complete the academic program, lack of exposure to anesthesiology, dishonesty/plagiarism, repeated poor grades, neutral or questionable reference letters, attitude problems identified, and disorganized application. The aforementioned factors were assessed using a five-point Likert scale, categorized as follows: absolutely important, slightly important, neutral, slightly unimportant, and absolutely unimportant.

The last section covered the proportion of the overall applicant score that the participant assigned to the following parameters: file review score (GPA), Saudi Medical License Examination (SMLE) score, curriculum vitae (CV), and interview score.

Statistical analysis

Statistical analysis of the obtained data was performed using the IBM Statistical Package for the Social Sciences (SPSS) statistical software version 21.0 (IBM Inc., Armonk, New York) to predict the level of significance of the framed hypothesis. The categorical and quantitative variables were described using descriptive statistics (frequency, percentile, average, and standard deviation). Statistically significant correlation and integrity of the results were evaluated based on a p-value of ≤0.05.

## Results

**Figure 1:**
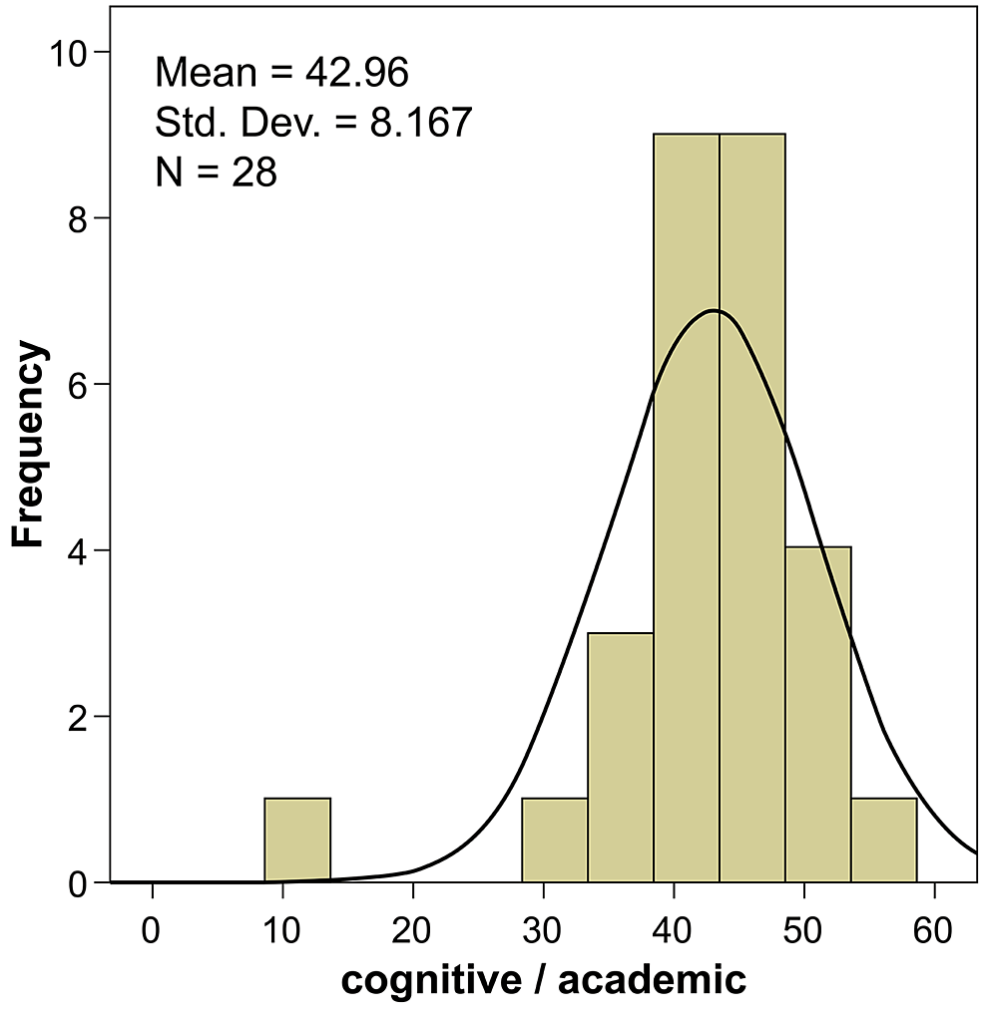
Evaluation ratings for cognitive/academic assessment measures

The first section of the survey focused on the assessment measures of the demographic data. The results obtained for the second section, which covered the cognitive/academic measures of the candidate, are included in Table 1. Elective experience in anesthesiology was given the highest degree of importance (90.71) among the considerations that determined the applicant’s selection to the anesthesiology residency program; 67.9% of experts considered it to be an absolutely important assessment measure. A master’s or doctorate degree was considered the least important (degree of importance: 67.86) among the 11 assessment measures; only 25% of experts considered it to be absolutely important. The remaining parameters received a neutral response from the participants regarding their importance for candidate assessment (Figure 1). 

**Table 1:** Cognitive/academic assessment measures in applicant evaluation for anesthesiology residency programs GPA - grade point average

			How do you rate the following cognitive/academic assessment measures in the evaluation of applicants to the anesthesiology residency program?					Degree of importance	Chi-square test	
			Absolutely unimportant	Slightly unimportant	Neutral	Slightly important	Absolutely important		X^2^	p-value
1	Medical school GPA	N	2	1	1	15	9	80.00	27.714	0.000
		%	7.1%	3.6%	3.6%	53.6%	32.1%			
2	Pre-clinical/pre-clerkship grades	N	1	2	4	14	7	77.14	19.500	0.001
		%	3.6%	7.1%	14.3%	50.0%	25.0%			
3	Clinical/clerkship grades	N	1	1	4	11	11	81.43	18.429	0.001
		%	3.6%	3.6%	14.3%	39.3%	39.3%			
4	Medical student transcript	N	2	3	5	9	9	74.29	7.714	0.103
		%	7.1%	10.7%	17.9%	32.1%	32.1%			
5	Academic awards	N	2	1	5	13	7	75.71	16.286	0.003
		%	7.1%	3.6%	17.9%	46.4%	25.0%			
6	Research experience/publications	N	1	1	5	13	8	78.57	18.429	0.001
		%	3.6%	3.6%	17.9%	46.4%	28.6%			
7	Master’s degree/Ph.D.	N	2	4	10	5	7	67.86	6.643	0.156
		%	7.1%	14.3%	35.7%	17.9%	25.0%			
8	Reference letters	N	1	2	5	11	9	77.86	13.429	0.009
		%	3.6%	7.1%	17.9%	39.3%	32.1%			
9	Elective experience in anesthesiology	N	1	0	1	7	19	90.71	30.857	0.000
		%	3.6%	0.0%	3.6%	25.0%	67.9%			
10	Medical school reputation	N	1	2	5	11	9	77.86	13.429	0.009
		%	3.6%	7.1%	17.9%	39.3%	32.1%			
11	Saudi Medical Licensing Exam (SMLE)	N	2	0	6	11	9	77.86	6.571	0.087
		%	7.1%	0.0%	21.4%	39.3%	32.1%			

There were no significant associations between medical student transcript (p=0.103), master’s or doctorate degree (p=0.156), and passing the Saudi Medical License Examination (SMLE), indicating that the experts’ answers were diverse, and no particular rating could be suggested for these parameters. The average ratings of all cognitive/academic measures are shown in Table 2; the overall ratings were higher when considering the cognitive/academic measures of the candidate for the assessment.

**Table 2:** Overall ratings of the cognitive/academic assessment measures

Cognitive/academic assessment measures	N	%	Score	
			Range	Mean±SD
Weak	1	3.6	11-54	42.964±8.167
Average	8	28.6		
High	19	67.9		
Total	28	100		

**Figure 2:**
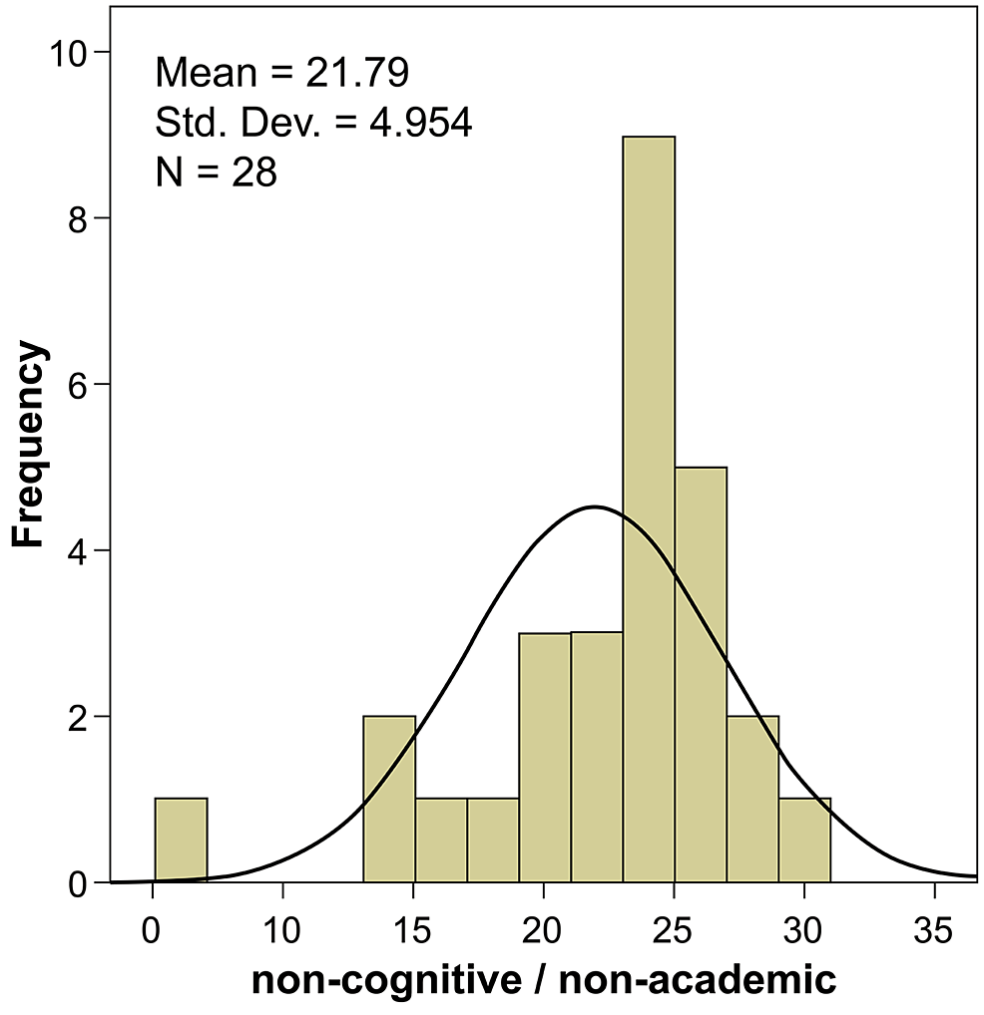
Evaluation ratings for non-cognitive/non-academic assessment measures

The third section included six assessment measures for candidate evaluation, among which work experience was considered an absolutely important parameter by ~43% of experts (degree of importance: 79.29). Extracurricular activities in medical school and leadership positions held were considered equally important for candidate selection (degree of importance: 76.43). Conduct on social media was the least considered measure among the six (degree of importance: 59.29). Table 3 shows the non-cognitive/non-academic assessment measures and the ratings for each measure by the experts and the statistical analysis of the data obtained. Applicant contact with the program director/colleagues was not a significant parameter, despite a degree of importance of 70.71, indicating the varied opinions of the experts regarding this measure. Table 4 shows the overall ratings of the non-cognitive/non-academic measures; ~61% of the experts strongly recommended these measures for the assessment (Figure 2).

**Table 3:** Non-cognitive/non-academic assessment measures in the evaluation of applicants to anesthesiology residency programs

			How do you rate the following non-cognitive/non-academic assessment measures in the evaluation of applicants to the anesthesiology residency program?					Degree of importance	Chi-square test	
			Absolutely unimportant	Slightly unimportant	Neutral	Slightly important	Absolutely important		X^2^	p-value
1	Extracurricular activities in medical school	N	1	1	5	16	5	76.43	27.000	0.000
		%	3.6%	3.6%	17.9%	57.1%	17.9%			
2	International/community service	N	1	0	9	15	3	73.57	17.143	0.001
		%	3.6%	0.0%	32.1%	53.6%	10.7%			
3	Leadership positions held	N	2	1	5	12	8	76.43	14.500	0.006
		%	7.1%	3.6%	17.9%	42.9%	28.6%			
4	Conduct on social media	N	5	3	12	4	4	59.29	9.500	0.050
		%	17.9%	10.7%	42.9%	14.3%	14.3%			
5	Applicant contact with program director/colleagues	N	3	3	6	8	8	70.71	4.500	0.343
		%	10.7%	10.7%	21.4%	28.6%	28.6%			
6	Work experience	N	1	3	4	8	12	79.29	13.786	0.008
		%	3.6%	10.7%	14.3%	28.6%	42.9%			

**Table 4:** Overall ratings of the non-cognitive/non-academic assessment measures

Non-cognitive/non-academic assessment measures	N	%	Score	
			Range	Mean±SD
Weak	3	10.7	6-29	21.786±4.954
Average	8	28.6		
High	17	60.7		
Total	28	100		

**Figure 3:**
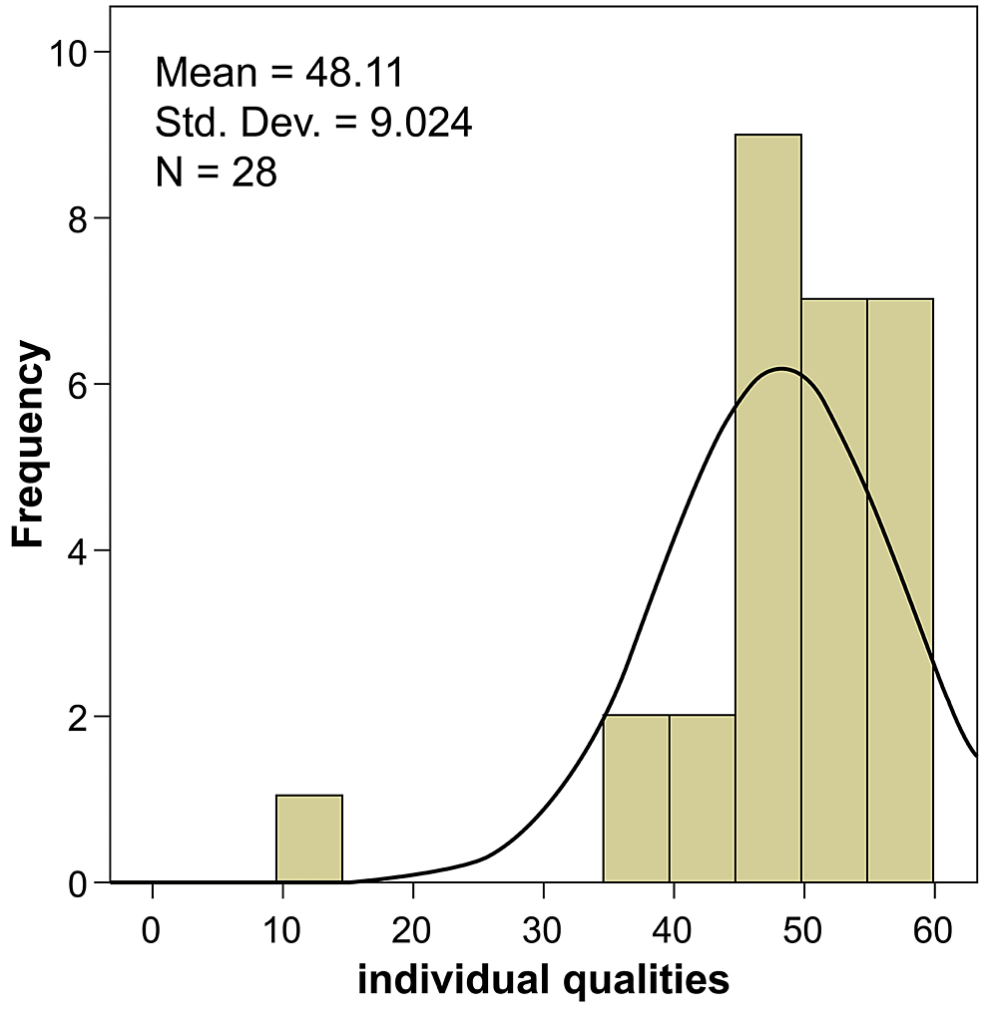
Evaluation ratings for individual qualities assessment measures

Section four covered approximately 14 parameters for candidate assessment, and the results are shown in Table 5. All parameters were found to be highly important, with the highest degree of importance being ≥82.14. Professionalism and the ability to think critically had the highest degree of importance (95.00) among the assessment measures; 75-86% of the experts regarded these measures to be absolutely important for candidate selection. Maturity was also observed to have almost equal importance (degree of importance: 94.29), as 86% of experts rated it as absolutely important. All 14 parameters were found to be highly significant (p≤0.05) during the statistical analysis. The overall ratings for individual qualities by the experts strongly suggested that individual qualities were given moderate priority with an average rating of 53.6% (Table 6, Figure 3).

**Table 5:** Individual qualities assessment measures in the evaluation of applicants to anesthesiology residency programs

			How do you rate the following individual qualities in evaluating applicants to the anesthesiology residency program?					Degree of importance	Chi-square	
			Absolutely unimportant	Slightly unimportant	Neutral	Slightly important	Absolutely important		X^2^	p-value
1	Professionalism	N	1	0	0	3	24	95.00	34.786	0.000
		%	3.6%	0.0%	0.0%	10.7%	85.7%			
2	Leadership skills	N	1	0	2	13	12	85.00	17.429	0.001
		%	3.6%	0.0%	7.1%	46.4%	42.9%			
3	Ability of critical thinking	N	1	0	0	3	24	95.00	34.786	0.000
		%	3.6%	0.0%	0.0%	10.7%	85.7%			
4	Ability to communicate efficiently	N	1	0	1	5	21	92.14	38.857	0.000
		%	3.6%	0.0%	3.6%	17.9%	75.0%			
5	Interpersonal skills	N	1	0	0	8	19	91.43	17.643	0.000
		%	3.6%	0.0%	0.0%	28.6%	67.9%			
6	Maturity]	N	1	0	1	2	24	94.29	55.143	0.000
		%	3.6%	0.0%	3.6%	7.1%	85.7%			
7	Motivation	N	1	0	1	4	22	92.86	43.714	0.000
		%	3.6%	0.0%	3.6%	14.3%	78.6%			
8	Confidence	N	1	0	0	7	20	92.14	20.214	0.000
		%	3.6%	0.0%	0.0%	25.0%	71.4%			
9	Altruism/selflessness	N	2	0	1	13	12	83.57	17.429	0.001
		%	7.1%	0.0%	3.6%	46.4%	42.9%			
10	Attitude	N	1	0	0	4	23	94.29	30.500	0.000
		%	3.6%	0.0%	0.0%	14.3%	82.1%			
11	Charismatic attributes	N	2	0	3	11	12	82.14	11.714	0.008
		%	7.1%	0.0%	10.7%	39.3%	42.9%			
12	Dexterity	N	2	0	2	13	11	82.14	14.571	0.002
		%	7.1%	0.0%	7.1%	46.4%	39.3%			
13	Quality of answers and questions	N	1	0	1	13	13	86.43	20.571	0.000
		%	3.6%	0.0%	3.6%	46.4%	46.4%			
14	Composure in interview	N	1	0	2	12	13	85.71	17.429	0.001
		%	3.6%	0.0%	7.1%	42.9%	46.4%			

**Table 6:** Overall ratings of the individual qualities assessment measures

Individual qualities	N	%	Score	
			Range	Mean±SD
Weak	1	3.6	12-58	48.107±9.024
Average	12	42.9		
High	15	53.6		
Total	28	100		

**Figure 4:**
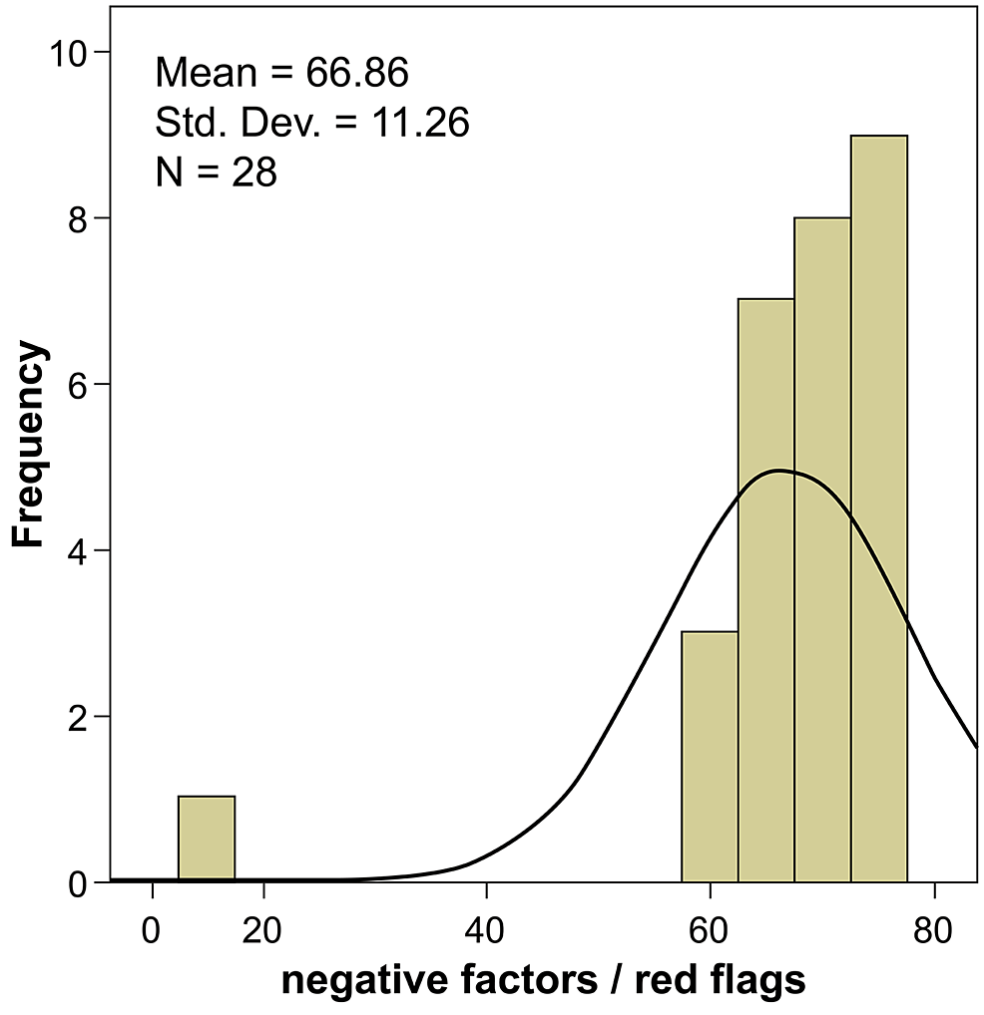
Evaluation ratings for negative factors and red flags assessment measures

Section five focused on the negative characteristics of individuals and the red flags associated with them during the selection process. Around 13 negative characters and red flags were considered and evaluated for priority during applicant selection (Table 7). Among these, attitude problems mentioned in the reference letter or observed during the interview were given higher priority and considered absolutely important by ~82% of the experts (degree of importance: 94.29). The absence of extracurricular activities was considered the least important measure; only 7% of experts considered it to be absolutely important (degree of importance: 66.43). Other characteristics such as poor literacy or communication skills, dishonesty, plagiarism, receiving disciplinary action in medical school, neutral or questionable reference letters were also considered to be highly important measures (degree of importance: 85.00-92.14). Except for delayed entry into residency after graduation, the remaining 12 parameters were statistically significant (p≤0.05). Table 8 and Figure 4 show the overall ratings for the negative factors and red flags.

**Table 7:** Negative factors and red flags assessment measures in evaluation of applicants to the anesthesiology residency program

			How do you rate the following negative factors/red flags in the evaluation of applicants?					Degree of importance	Chi-square	
			Absolutely unimportant	A bit unimportant	Neutral	Somewhat important	Absolutely important		X^2^	p-value
1	Received disciplinary action in medical school	N	2	0	4	5	17	85.00	19.714	0.000
		%	7.1%	0.0%	14.3%	17.9%	60.7%			
2	Delayed entry into residency after graduation	N	5	2	6	11	4	65.00	8.071	0.089
		%	17.9%	7.1%	21.4%	39.3%	14.3%			
3	Failure in a required clerkship rotation	N	3	1	2	15	7	75.71	23.429	0.000
		%	10.7%	3.6%	7.1%	53.6%	25.0%			
4	Failure in a pre-clinical course	N	3	1	3	15	6	74.29	22.000	0.000
		%	10.7%	3.6%	10.7%	53.6%	21.4%			
5	No extracurricular activities	N	3	1	10	12	2	66.43	18.071	0.001
		%	10.7%	3.6%	35.7%	42.9%	7.1%			
6	Poor literacy or communication skills	N	1	0	1	6	20	91.43	34.571	0.000
		%	3.6%	0.0%	3.6%	21.4%	71.4%			
7	Extended time needed to complete program for academic reasons]	N	1	2	4	12	9	78.57	15.929	0.003
		%	3.6%	7.1%	14.3%	42.9%	32.1%			
8	Lack of exposure to anesthesia (<4 weeks on rotation/elective)	N	2	2	3	17	4	73.57	29.500	0.000
		%	7.1%	7.1%	10.7%	60.7%	14.3%			
9	Dishonesty/plagiarism	N	1	1	0	4	22	92.14	43.714	0.000
		%	3.6%	3.6%	0.0%	14.3%	78.6%			
10	Repeated poor grades	N	2	1	2	9	14	82.86	23.071	0.000
		%	7.1%	3.6%	7.1%	32.1%	50.0%			
11	Neutral or questionable reference letter	N	2	0	3	7	16	85.00	17.429	0.001
		%	7.1%	0.0%	10.7%	25.0%	57.1%			
12	Attitude problems identified (reference letter, interview)	N	1	0	0	4	23	94.29	30.500	0.000
		%	3.6%	0.0%	0.0%	14.3%	82.1%			
13	Disorganized application	N	1	2	1	12	12	82.86	24.500	0.000
		%	3.6%	7.1%	3.6%	42.9%	42.9%			

**Table 8:** Overall ratings of the red flags and negative factors assessment measures

Negative Factors and red flags assessment measures	N	%	Score	
			Range	Mean ± SD
Weak	1	3.6	15-75	66.857±11.260
Average	0	0		
High	27	96.4		
Total	28	100		

The overall assessment of all measures considered for the study is shown in Table 9. The obtained results suggested that non-cognitive/non-academic, along with cognitive/academic measures, were significantly important for candidate selection, whereas negative factors and red flags were considered important even when cognitive/academic and non-cognitive/non-academic measures were good.

**Table 9:** Overall statistical analysis of all assessment measures

Assessment measures		Cognitive/academic	Non-cognitive/non-academic	Negative factors/red flags
Non-cognitive/non-academic	r	0.578		
	p-value	0.001*		
Negative factors/red flags	r	0.681	0.507	
	p-value	<0.001*	0.006*	
Individual qualities	r	0.776	0.553	0.812
	p-value	<0.001*	0.002*	<0.001*

## Discussion

The present study appeared to resolve the factors and assessment measures considered during candidate selection for anesthesiology residency programs in Saudi Arabia. A total of 28 experts were included in the study, and their original responses were recorded anonymously. The data obtained and analyzed revealed that the board of members and experts involved in the process of selecting applicants considered a variety of parameters when accepting or rejecting candidates. The questionnaire had a total of approximately 44 parameters, categorized into five ratings ranging from absolutely unimportant to absolutely important.

Significant factors such as the medical school’s GPA requirements, examination results, research activities, the training facility’s credibility, training period, workload and job duties, and the training atmosphere have a significant impact on program selection by applicants [11]. According to Orbach-Zinger et al., the percentage of medical students interested in anesthesiology as a specialty in the United States was 13%, compared to 0% in Israel, due to superior working circumstances and compensation [12]. In a population-based survey among undergraduate students in Saudi Arabia, the interest and willingness to work in the field of anesthesiology was less than 1%, which is extremely low and needs to be improved [13]. Hence, we evaluated the factors associated with applicant selection in the anesthesiology residency program in Saudi Arabia to encourage the applicants to overcome their difficulties in selecting their choice of medical specialty as well as increase their possibility of choosing anesthesiology.

To be eligible for a professional academic position in Saudi Arabia, most universities expect overseas credentials. Research activities are allocated 20 points during the selection of candidates for the residency program [14]. In the current survey, we observed that around 75% of experts considered research experience and publications to be important for applicant selection, which corresponded to the findings of previous reports.

In our survey results, relative experience in the field of anesthesiology was considered highly important among all cognitive/academic assessment measures during the selection of an anesthesiology residency program. Similarly, a few studies have reported that personal considerations are not constant and that they may change over time when a person is exposed to various factors, such as experiences and environmental circumstances [3, 11]. Those who wanted to pursue fellowship training and research following their residency reported improving their employability as the second most important criterion. Several studies have shown that gaining more experience improves one’s employability [6,15]. Acute or chronic pain management, regional anesthesia, simulation, and pediatric anesthesia are the four most common anesthesia subspecialties. Dedicated acute pain and regional anesthetic treatments are crucial for improving acute pain management, which can improve medical results and patient satisfaction and save costs. This would encourage healthcare providers to hire anesthesiologists with complete training in this field of study [16].

Research on the personal characteristics that are most suited to the field of anesthesiology has shown that anesthesiologists in New Zealand regarded individuality, regularity, and empathy as vital qualities in an anesthetist, but those in Scotland considered practicality as significant [17]. The current results also showed individual qualities to be the most important traits among the selection criteria for applicants. Characteristics such as professionalism, leadership skills, critical thinking ability, communication efficiency, interpersonal skills, maturity, motivation, confidence, and attitude were deemed important in the selection process of anesthetists.

Lifestyle habits have not been considered a major criterion for candidate selection [18], and it was of the least importance in the current survey. Similar studies on the selection criteria for anesthesiology residents in the United States have reported that education from an American medical school, USMLE step two scores, relatively young age, and female gender are considered favorable factors for applicant selection [8]. In contrast, the current survey showed that demographic factors such as age and gender did not influence the selection criteria in Saudi Arabia; however, the SMLE scores were considered important for the selection of applicants by 71% of the experts.

In Canadian anesthesiology programs, applicants with high academic achievement, expertise in elective anesthetic procedures, genuineness, and broad activities and interests are given priority during the selection process [19]. The study also added that the candidates’ reference letters and personal relationships were noteworthy during the selection process [19]; this was in partial contrast to the results of our study. According to our study participants, reference letters mentioning the attitude of the applicant were considered important, though a personal relationship with the board members was not considered as important. Other assessment measures such as academic achievement, expertise in elective anesthetic procedures, genuineness, broad activities, and red flags and negative factors for candidate selection observed in our study were positively correlated with the previously reported factors [19].

Unfortunately, only a limited number of studies have been published on the factors and assessment measures associated with applicant selection for anesthesiology residency programs in Saudi Arabia. Hence, our study presents a novel identification of approximately 44 factors that may be considered during the selection process and ranks them from highest to lowest priority. This would help the students brace themselves for the selection process of the anesthesia residency program in Saudi Arabia.

Although the current study was conducted with a wide range of questions and anonymous answers, it has the following limitations: we included a small sample size of only 28 participants. Further, the study did not include any applicants who had been previously selected or rejected from a residency program, which would have allowed us to cross-check the obtained results; nonetheless, we do not believe this would have affected the results of our analysis.

## Conclusions

Based on the current findings, it can be suggested that both cognitive/academic characteristics and non-academic/non-cognitive characteristics, including individual characteristics of the applicant, are considered important during the selection process for the anesthesiology residency program in Saudi Arabia. Students should be aware of red flags and negative factors, such as attitude, dishonesty, and poor communication skills. These should be avoided, which can increase their chances to be selected during the admission process. We recommend that students take electives in anesthesia and maintain a high level of professionalism. In addition, maturity and the ability to critically think help in opting for the specialty.
